# Exposure-based stratification of postoperative complications in major cardiac surgical procedures: a retrospective cohort study using the MOVER database

**DOI:** 10.3389/fcvm.2026.1887472

**Published:** 2026-09-21

**Authors:** Ruining Li

**Affiliations:** Department of Surgery, Beijing Anzhen Hospital, Capital Medical University, Beijing, China

**Keywords:** aortic surgery, coronary artery bypass, perioperative complications, perioperative risk, surgical exposure

## Abstract

**Objective:**

Postoperative complications and perioperative physiologic stress remain major contributors to postoperative morbidity following complex cardiac surgery. This study aimed to evaluate perioperative exposure patterns and develop an exploratory exposure-based risk stratification framework for examining associations with postoperative complications among patients undergoing coronary artery bypass grafting (CABG) and major aortic surgical procedures using the MOVER database.

**Methods:**

A retrospective cohort study was conducted using the EPIC_EMR component of the MOVER perioperative database. Among 65,728 surgical records, 639 major cardiac surgical procedures, including CABG and aortic procedures, from 609 unique patients were identified after applying inclusion criteria and data quality filtering. Baseline demographic characteristics, perioperative exposure variables, and diagnostic history were integrated for analysis. Logistic regression modeling was used to identify variables associated with postoperative complications. A composite perioperative exposure score (incorporating operating room duration, anesthesia duration, and diagnosis burden) was created to classify patients into low, intermediate, and high-exposure groups.

**Results:**

The median patient age was 64 years (IQR 55.5–71.0), and 77.5% of patients were male. Median operating room and anesthesia durations were 423.0 min (IQR 374.0–487.0) and 437.0 min (IQR 389.0–505.0), respectively. Overall, postoperative complications occurred in 23 of 639 procedures (3.6%). In multivariable analysis, anesthesia duration (OR 1.006, 95% CI 1.002–1.010, *p* = 0.005) and operating room duration (OR 1.004, 95% CI 1.001–1.008, *p* = 0.021), remained associated with postoperative complications, whereas hospital length of stay was analyzed separately as a postoperative outcome rather than included as a predictor. Patients in the highest exposure tertile demonstrated 2.31-fold higher odds of complications (95% CI 1.12–4.76, *p* = 0.023) compared with the lowest exposure group.

**Conclusion:**

Prolonged perioperative exposure is significantly associated with postoperative complications in major cardiac surgical procedures. Exposure-based perioperative metrics may serve as practical indicators of perioperative exposure burden and provide an exploratory framework for risk assessment in cardiac surgical populations.

## Introduction

Perioperative physiologic instability and postoperative complications remain a major threat to organ perfusion during and after cardiac surgery (1). In major cardiac procedures, including coronary artery bypass grafting (CABG) and complex aortic surgery, cardiopulmonary bypass, myocardial ischemia–reperfusion injury, systemic inflammation, bleeding, and changing vasoactive requirements contribute to perioperative risk (2). Consequently, assessment of procedural complexity and perioperative exposure may complement conventional baseline risk assessment.

Perioperative hypotension is clinically important because exposure to mean arterial pressure (MAP) below commonly used thresholds is associated with an increased risk of myocardial and kidney injury, particularly when hypotension is prolonged or severe (3). However, hemodynamic optimization studies have reported variable clinical benefits, highlighting the need for context-specific approaches to perioperative risk assessment (4, 5).

Patients undergoing CABG and aortic surgery are particularly vulnerable to CPB-related vasoplegia and post-cardiotomy circulatory failure, which are associated with increased postoperative morbidity and mortality (6). Given the multifactorial nature of perioperative physiologic stress, risk assessment should consider procedural complexity and perioperative exposure alongside baseline clinical factors and established cardiac surgery-specific predictors where available.

Recent advances in machine learning applied to physiologic time series suggest that high-resolution monitoring data can support early prediction of circulatory deterioration (7). Arterial waveform models have predicted MAP <65 mmHg up to about 15 min before hypotensive events (8), while transformer-based sequence models incorporating vital signs, medications, and ventilation variables have been investigated for prediction of intraoperative blood pressure (9). However, access to sufficiently complete high-resolution physiologic data may be limited, creating a need to examine whether routinely available perioperative EMR variables can provide complementary information regarding exposure burden and postoperative outcomes. Such EMR-derived variables should be regarded as surrogate indicators of perioperative exposure rather than direct measures of physiologic instability.

The Medical Informatics Operating Room Vitals and Events Repository (MOVER) contains linked electronic medical record (EMR) data and high-fidelity intraoperative physiologic data from a large surgical population (10). The present study specifically used the EPIC_EMR component because it contained the structured demographic, diagnostic, procedural, anesthesia, hospitalization, and postoperative complication variables required to construct the cardiac surgical cohort and evaluate the predefined exposure–outcome associations. High-resolution waveform and time-series data were not incorporated into the present analysis; accordingly, this study evaluates aggregated EMR-derived exposure variables rather than a dynamic physiologic prediction model.

In the present study, we used the MOVER EPIC_EMR dataset to examine associations between routinely available perioperative exposure variables—including operating room duration, anesthesia duration, and diagnosis burden—and documented postoperative complications following major cardiac surgical procedures. Operating room duration and anesthesia duration were considered surrogate measures of procedural exposure rather than direct measures of perioperative physiology. The primary endpoint was the occurrence of at least one documented postoperative complication during the index hospitalization. Secondary objectives were to evaluate multivariable exposure–outcome associations and explore the discrimination and calibration of an exposure-based framework. Given the retrospective design and use of aggregated EMR variables, the framework was considered exploratory rather than a validated clinical prediction tool.

## Methodology

### Study design and data source

This study was conducted to examine associations between perioperative exposure variables and postoperative complications in patients undergoing complex cardiovascular surgical procedures. Data were obtained from the Medical Informatics Operating Room Vitals and Events Repository (MOVER), a publicly available perioperative database containing de-identified electronic medical records (EMRs), intraoperative physiologic monitoring data, medication records, and clinical outcomes from surgical patients treated at the University of California, Irvine Medical Center. The present analysis specifically used the EPIC_EMR component because it contained the structured demographic, diagnostic, procedural, anesthesia, hospitalization, and postoperative complication variables required for cohort construction and the planned exposure–outcome analyses. High-resolution waveform and time-series data were not incorporated into the present analysis. Accordingly, the present analysis used aggregated EMR-derived surrogate exposure variables rather than direct time-resolved perioperative physiologic measures.

### Study population and cohort selection

The surgical procedure descriptions and procedural codes present in the dataset were used to identify patients who underwent CABG and aortic procedures in the EMR tables. The analytic cohort included CABG procedures, aortic procedures, and other major cardiac procedures identified from the EMR tables, rather than exclusively combined CABG and aortic surgeries. All adult patients aged 18 years and older with the required EPIC_EMR perioperative and clinical records were eligible for inclusion. Cases with missing data on key exposure variables or information required to ascertain postoperative complications were excluded. Moreover, duplicated records and entries with conflicting or erroneous information were removed at the stage of data preprocessing to ensure the trustworthiness and quality of the study data. The final cohort comprised patients whose perioperative clinical and procedural information was available for analysis after the application of these inclusion and exclusion criteria.

### Data extraction and variable selection

Relevant demographic, diagnostic, procedural, and outcome variables were extracted from the EPIC_EMR component of MOVER. Baseline characteristics included age, sex, and body mass index. The variables used in the present analysis included age, sex, diagnosis burden, operating room duration, anesthesia duration, and hospital length of stay. Hospital length of stay was extracted solely for descriptive postoperative analyses and was not incorporated into the multivariable regression model or composite exposure score because it is determined after surgery and may be influenced by postoperative complications. Thus, hospital length of stay did not contribute to the exposure score or risk estimation, avoiding its use as a predictor of an outcome that could itself influence length of stay. These variables were selected based on their availability within the EPIC_EMR component of the MOVER database and their reported clinical relevance to perioperative outcomes. Important cardiac surgery–specific variables, including cardiopulmonary bypass time, aortic cross-clamp time, left ventricular ejection fraction (LVEF), urgency of surgery, and validated surgical risk scores (e.g., STS or EuroSCORE), were not consistently available in the extracted dataset and therefore could not be incorporated into the present analysis. Consequently, the present analysis could not determine whether the observed associations were independent of these established cardiac surgery-specific risk factors. Procedure type (CABG, valve surgery, ascending aortic repair, thoracic endovascular aortic repair (TEVAR), and other cardiac procedures) was also extracted to characterize the procedural heterogeneity of the study cohort.

### Outcome definition

The primary outcome was a composite binary endpoint defined as the occurrence of at least one documented postoperative complication during the index hospitalization. Postoperative complications were identified using database fields designated for postoperative complication documentation linked to each surgical procedure. The observation period extended from completion of the index cardiac surgical procedure until discharge from the corresponding hospitalization, consistent with the complication records available within the EPIC_EMR database.

The complication category included cardiovascular complications (e.g., circulatory or cardiac events) and other documented postoperative adverse events available in the database. Multiple complication entries occurring during the same hospitalization were treated as a single binary outcome (presence versus absence of any postoperative complication) for regression analyses.

Because this study relied on routinely collected EMR data, no additional manual chart review or independent clinical adjudication was performed. To improve data reliability, duplicate complication records linked to the same procedure were removed during preprocessing, and complication entries were cross-checked against unique procedure identifiers to ensure correct linkage between surgical episodes and postoperative records. Consequently, complication ascertainment depended on the completeness and accuracy of database coding and should be interpreted within this context.

### Data preprocessing

Data cleaning procedures were performed to eliminate duplicate records and correct inconsistencies in measurement units and variable formats. The extent and pattern of missing data were assessed for all study variables before analysis to determine whether missingness was limited and suitable for simple imputation. Variables with only a small proportion of missing values (<5%) were imputed using simple statistical methods according to variable type, with median imputation for continuous variables and mode imputation for categorical variables. Cases with missing values in key outcome variables or essential perioperative exposure variables were excluded during cohort selection. Following imputation and exclusion of incomplete records, all variables included in the multivariable analyses were complete for the final analytic cohort.

### Model development

Logistic regression modeling was used to evaluate associations between perioperative exposure variables and documented postoperative complications rather than to develop a purely predictive preoperative risk model. The exposure variables were treated as surrogate measures of procedural exposure and were not considered direct measures of perioperative physiologic instability. Clinically relevant variables were selected based on prior literature and data availability. The multivariable model included demographic characteristics (age and sex), diagnosis burden, operating room duration, anesthesia duration, and ICU admission. Hospital length of stay was intentionally excluded from the multivariable model because it is a postoperative measure that may be influenced by the occurrence of complications; it was evaluated separately as a descriptive recovery outcome. Cardiopulmonary bypass time, aortic cross-clamp time, LVEF, operative urgency, and STS/EuroSCORE could not be included because these variables were not consistently available in the extracted dataset.

### Statistical analysis

Statistical analyses were performed using Python. Continuous variables were summarized as mean ± standard deviation when normally distributed and as median and interquartile range when non-normally distributed; categorical variables were summarized as frequencies and percentages. Continuous variables were compared using independent-samples t-tests or Mann–Whitney U tests, as appropriate, and categorical variables were compared using chi-square or Fisher's exact tests. Pairwise correlations among candidate continuous predictors were examined before multivariable modeling. Given the strong correlation observed between operating room duration and anesthesia duration (*r* = 0.91), multicollinearity was specifically assessed using variance inflation factors (VIFs). VIFs were calculated for the predictors included in the final multivariable model to quantify multicollinearity and assess its potential influence on coefficient stability. Model discrimination was evaluated using the area under the receiver operating characteristic (ROC) curve. Calibration was assessed by comparing predicted and observed complication risks across the analytic cohort using calibration plots. Exploratory classification performance was additionally summarized using sensitivity, specificity, positive predictive value (precision), negative predictive value, and F1-score at the prespecified classification threshold. Given the exploratory nature of the analysis and low number of outcome events, these metrics were interpreted cautiously and were not considered evidence of clinical validity. Effect estimates and model-performance measures are reported with 95% confidence intervals (CIs), where applicable. Statistical significance was defined as a two-sided *p*-value <0.05.

### Composite exposure score construction

To provide an overall measure of perioperative exposure burden, a composite exposure score was developed using variables selected on the basis of their clinical relevance to procedural and baseline exposure burden. These variables included operating room duration, anesthesia duration, and diagnosis burden. Hospital length of stay was excluded from the composite exposure score because it is measured after surgery and may be influenced by postoperative complications, making it inappropriate as a perioperative exposure variable for exposure stratification. Operating room duration and anesthesia duration were selected as surrogate measures of cumulative intraoperative exposure, whereas diagnosis burden was included to reflect baseline clinical complexity. These surrogate measures do not directly quantify intraoperative physiologic instability.

Each variable was standardized using z-score normalization before score construction to account for differences in measurement scales. Equal weighting was applied as a pragmatic approach to provide a simple and interpretable summary of exposure burden. However, the equal-weighting scheme was not derived from regression coefficients, statistical optimization, or established clinical weighting criteria and therefore does not imply equivalent prognostic importance of the individual components. Accordingly, the composite score should be regarded as an exploratory exposure index rather than a validated clinical risk score. Future studies should evaluate statistically derived or clinically informed weighting strategies and determine whether these approaches improve discrimination and clinical utility in independent cohorts incorporating direct physiologic measurements and established cardiac surgery-specific risk variables.

### Ethical considerations

The study used a publicly available and fully de-identified dataset and did not involve direct interaction with patients, nor did it involve access to identifiable personal information. Therefore, institutional ethical approval for human subject research was not required for this study. All analyses were performed following the policies of data use and ethical guidelines for secondary analysis of de-identified clinical data.

## Results

### Cohort selection and data integration

The EPIC_EMR surgical repository initially contained 65,728 perioperative records. Using procedure descriptions and surgical codes, cases corresponding to coronary artery bypass grafting (CABG) and major aortic surgical procedures were identified. Cases with missing values in essential study variables, including key perioperative exposure measures and postoperative outcome data, were excluded during cohort selection. Following removal of duplicate surgical log entries and verification of patient identifiers, the final analytic cohort comprised 639 surgical procedures from 609 unique patients. These records were then linked with patient history and diagnosis records using unique patient identifiers, enabling integration of baseline demographic data, comorbidity burden, and perioperative surgical data. The cohort derivation process is illustrated in Figure 1.

**Figure 1:**
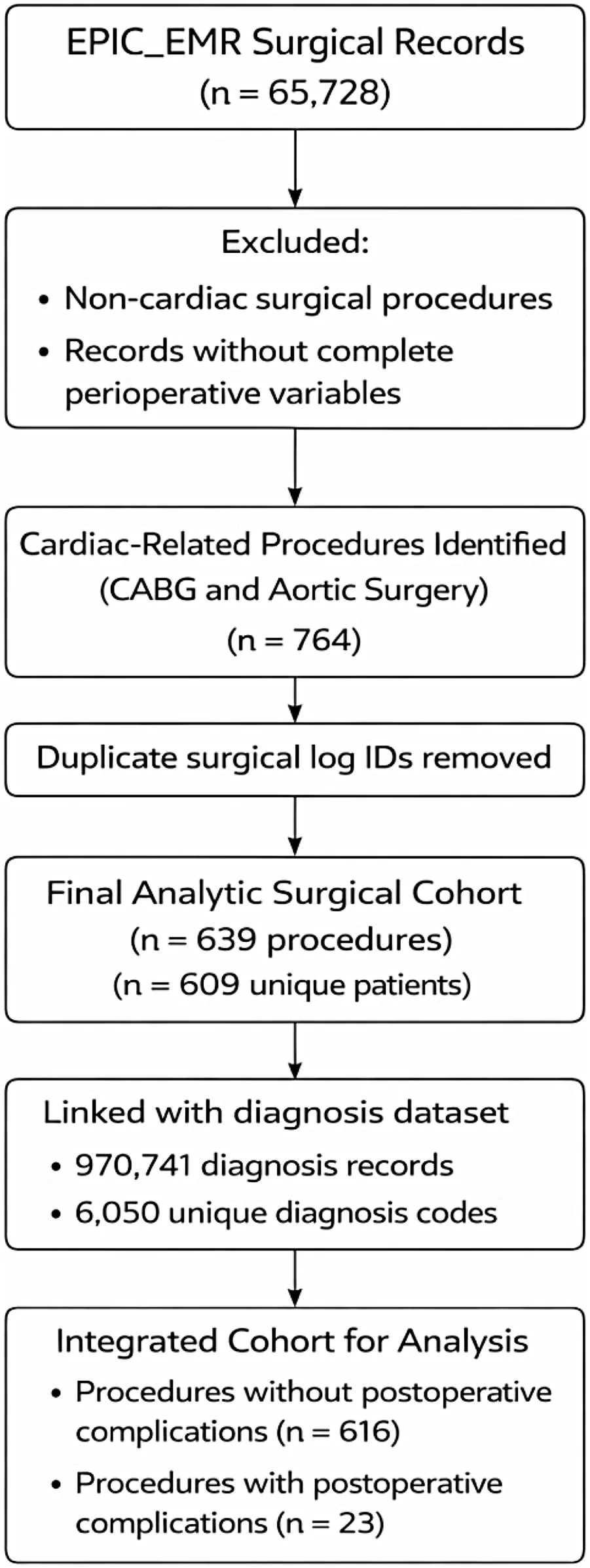
Flow diagram illustrating the identification of the cardiac surgical cohort from the EPIC_EMR database and integration with patient diagnostic records used for analysis of perioperative outcomes.

### Demographic and baseline clinical characteristics

Baseline characteristics of the cohort of cardiac surgical patients are shown in Table 1. The median age of the patients was 64 years (IQR 55.5-71.0). Male patients were in the majority (77.5% of the cohort, 495/639; 95% CI 74.3%–80.5%), and females 22.5% (95% CI 19.5%–25.7%). Perioperative procedural durations were prolonged. The median operating room duration was 423.0 min (IQR 374.0–487.0), and the median anesthesia duration was 437.0 min (IQR 389.0–505.0). Postoperative hospital length of stay, analyzed as a descriptive postoperative recovery outcome, showed moderate variability, with a median of 11.0 days (IQR 8.0–16.0). Postoperative intensive care utilization was nearly universal in this cohort, with 630 of 639 procedures requiring ICU admission (98.6%; 95% CI 97.4%–99.4%).

**Table 1:** Baseline demographic and perioperative characteristics of the cardiac surgical cohort.

Variable	Value*	95% CI
Procedures	639	–
Unique patients	609	–
Age (years), median (IQR)	64 (55.5–71.0)	–
Male sex, *n* (%)	495 (77.5%)	74.3–80.5
Female sex, *n* (%)	144 (22.5%)	19.5–25.7
Hospital length of stay (days), median (IQR)	11.0 (8.0–16.0)	–
Operating room duration (min), median (IQR)	423.0 (374.0–487.0)	–
Anesthesia duration (min), median (IQR)	437.0 (389.0–505.0)	–
ICU admission, *n* (%)	630 (98.6%)	97.4–99.4

*Values are reported as median (interquartile range) or number (percentage).

### Burden of preoperative comorbid conditions

Analysis of the linked diagnosis dataset revealed 970,741 diagnosis entries in 43,547 unique patients that represented 6,050 unique diagnostic codes (Table 2). At the patient level, the median number of diagnoses was 7 (IQR 2-20), suggesting substantial variability in baseline health status and chronic disease burden among patients undergoing cardiac surgery. However, the number of diagnoses represents a crude summary of comorbidity burden and should not be interpreted as equivalent to validated comorbidity indices. The most commonly documented comorbid conditions were hypertension, hyperlipidemia, anemia, vitamin D deficiency, and hypothyroidism, but acute clinical conditions such as acute kidney injury, sepsis, and others were also documented in a subset of patients. These findings indicate the presence of substantial baseline multimorbidity in the study cohort, which may increase susceptibility to adverse perioperative outcomes.

**Table 2:** Summary of diagnostic burden in the linked patient population.

Metric	Value
Total diagnosis records	970,741
Unique patients with diagnoses	43,547
Unique diagnosis codes	6,050
Median diagnoses per patient	7
Interquartile range	2–20

### Perioperative procedural exposure

Detailed perioperative metrics extracted from EPIC_EMR records demonstrated prolonged procedural durations in many cases. Table 3 presents the results indicating that median operating room and anesthesia durations exceeded 7 h, reflecting prolonged procedural exposure in this cardiac surgical cohort. The variation in hospital length of stay was also quite wide among patients, reflecting variability in postoperative recovery. The nearly universal ICU admission rate also highlights the fact that this surgical population requires substantial postoperative critical-care utilization.

**Table 3:** Perioperative characteristics of cardiac surgical procedures.

Variable	Median (IQR)
Operating room duration (min)	423.0 (374.0–487.0)
Anesthesia duration (min)	437.0 (389.0–505.0)
Hospital length of stay (days)	11.0 (8.0–16.0)
ICU admission (%)	98.6

Hospital length of stay is presented descriptively and was not included in the multivariable model or composite exposure index.

### Postoperative complication incidence and clinical associations

Overall, 23 out of 639 procedures (3.6%; 95% CI, 2.3%–5.4%) had at least one documented postoperative complication recorded in the database. Cardiovascular complications were recorded in 7 procedures (1.1%), comprising 4 circulatory/hemodynamic-related events (0.6%) and 3 other cardiovascular events (0.5%), including one arrhythmia-related event, one myocardial ischemic/cardiac event, and one other cardiac event. The remaining 16 procedures (2.5%) had non-cardiovascular complications, including respiratory/pulmonary (*n* = 5), renal (*n* = 3), bleeding/hematologic (*n* = 3), neurologic (*n* = 2), infectious (*n* = 2), and metabolic/other systemic complications (*n* = 1). A complete breakdown of the 23 procedures constituting the primary endpoint is provided in Supplementary Table S1. Comparison of the procedures with and without postoperative complications showed that there were a number of statistically significant differences (Table 4). Patients with postoperative complications had a longer hospital stay (median 14 vs. 11 days, *p* = 0.042) as well as longer operating room time (481.5 vs. 422.0 min, *p* = 0.030) and anesthesia time (512.0 vs. 434.0 min, *p* = 0.007). Hospital length of stay was considered a descriptive postoperative recovery outcome and not a predictor of complications. No significant differences were observed in demographic characteristics such as age or sex distribution. The observed complication rate was lower than expected for high-risk cardiac surgery and may reflect underreporting, incomplete capture, differences in complication coding within the database.

**Table 4:** Comparison of procedures with and without postoperative complications.

Variable	With complication (*n* = 23)	Without complication (*n* = 616)	*p*-value
Age (years)	65.0 (57.0–76.0)	63.0 (55.0–71.0)	0.444
Male sex (%)	82.6	77.3	0.799
Postoperative length of stay (days)	14.0 (11–21)	11.0 (8–16)	0.042
OR duration (min)	481.5 (425–572)	422.0 (373–485)	0.030
Anesthesia duration (min)	512.0 (445–621)	434.0 (388–502)	0.007
ICU admission (%)	100	98.5	1.000

Postoperative length of stay was analyzed as a descriptive recovery outcome and was not included as a predictor in the multivariable model or composite exposure index.

### Perioperative exposure stratification

To explore perioperative factors associated with postoperative complications, an exposure-based stratification framework was developed using available perioperative exposure variables. As continuous physiologic time-series data were not incorporated, surrogate measures of perioperative exposure, such as procedure length, exposure to anesthesia, and comorbidity burden, were considered to examine associations with adverse outcomes. Accordingly, these variables represent surrogate indicators of procedural exposure rather than direct measures of perioperative physiologic instability. Univariate comparisons also revealed that longer perioperative exposure times were associated with postoperative adverse events. Median operating room duration was 481.5 min in cases with complications compared with 422.0 min in uncomplicated procedures (*p* = 0.030). Similarly, the duration of anesthesia exposure was higher in the group with complications (512.0 vs. 434.0 min, *p* = 0.007). There was also increased postoperative hospitalization in patients with complications (14.0 vs. 11.0 days, *p* = 0.042). Because hospital length of stay occurs after surgery, this difference was considered descriptive and was not incorporated into the exposure score or multivariable association framework. These results indicate that prolonged operative duration is associated with increased likelihood of postoperative complications, although causal relationships cannot be inferred from this analysis. The distribution of the perioperative exposure variables and their association with the occurrence of complications are shown in Figure 2.

**Figure 2:**
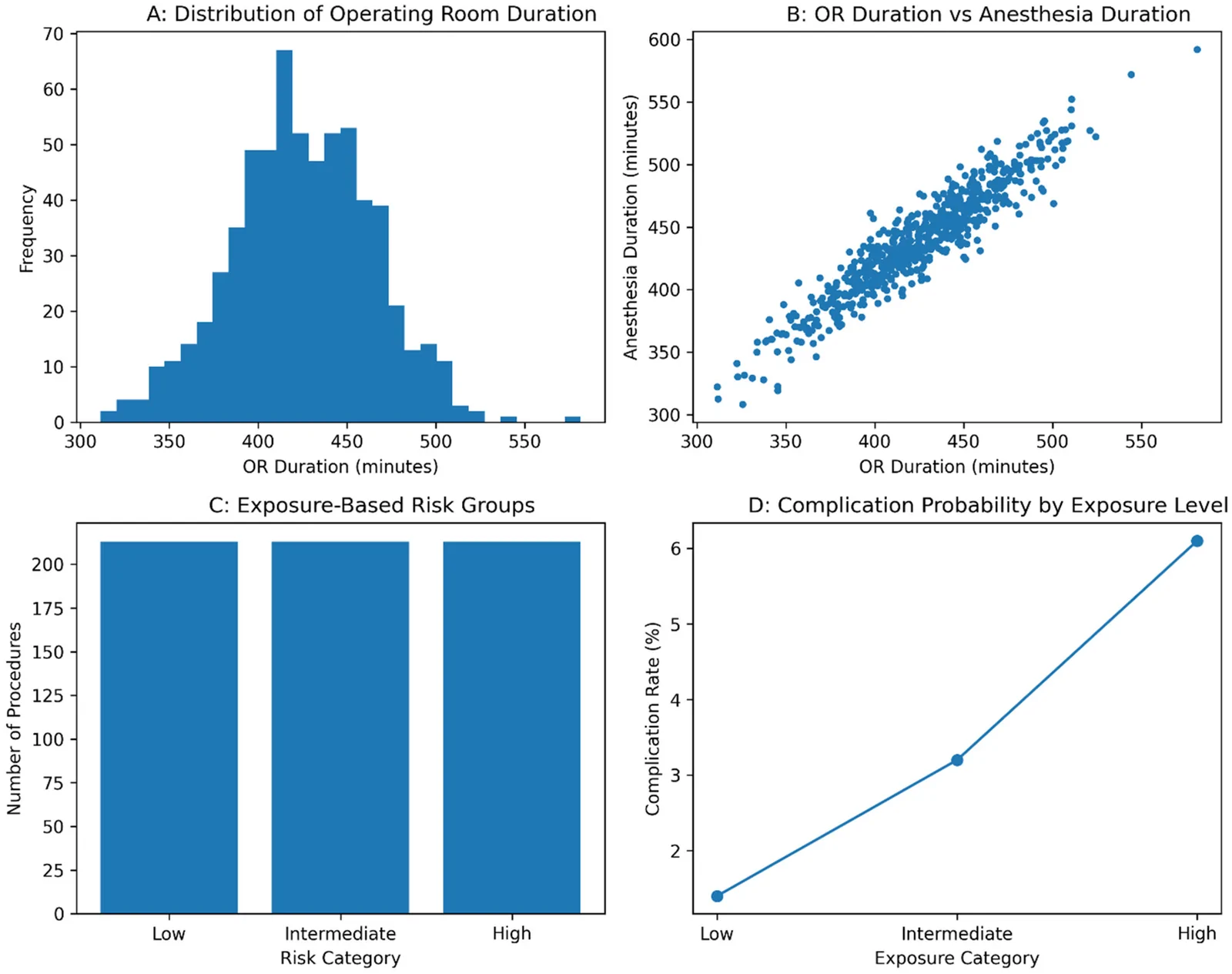
Exposure-based patterns of perioperative procedural burden and postoperative complication occurrence. **(A)** Distribution of operating room duration across cardiac surgical procedures. **(B)** Relationship between operating room duration and anesthesia duration. **(C)** Distribution of procedures across exposure-based categories derived from perioperative exposure variables. **(D)** Observed postoperative complication rates across exposure strata.

### Multivariable associations with postoperative complications

A multivariate logistic regression model was developed to evaluate associations between perioperative variables and postoperative complications rather than to develop a purely predictive preoperative risk model. The model included demographic factors (age and sex), the perioperative exposure factors (operating room time and anesthesia time), and comorbidity burden (number of diagnoses). Hospital length of stay was not included in the multivariable model because it is measured after surgery and may itself be influenced by postoperative complications. Figure 3A presents the adjusted odds ratios and 95% confidence intervals for all variables included in the multivariable model. As Table 5 shows, anesthesia duration showed the strongest association with postoperative complications (OR 1.006, 95% CI 1.002-1.010, *p* = 0.005). Operating room duration was also significantly associated with postoperative complications (OR 1.004, 95% CI 1.001-1.008, *p* = 0.021). Multicollinearity diagnostics demonstrated elevated VIFs for operating room duration (VIF = 5.82) and anesthesia durations (VIF = 6.14), consistent with the strong correlation between these variables (*r* = 0.91). Overall, operative and anesthesia durations were the principal variables associated with postoperative complications in the adjusted model.

**Figure 3:**
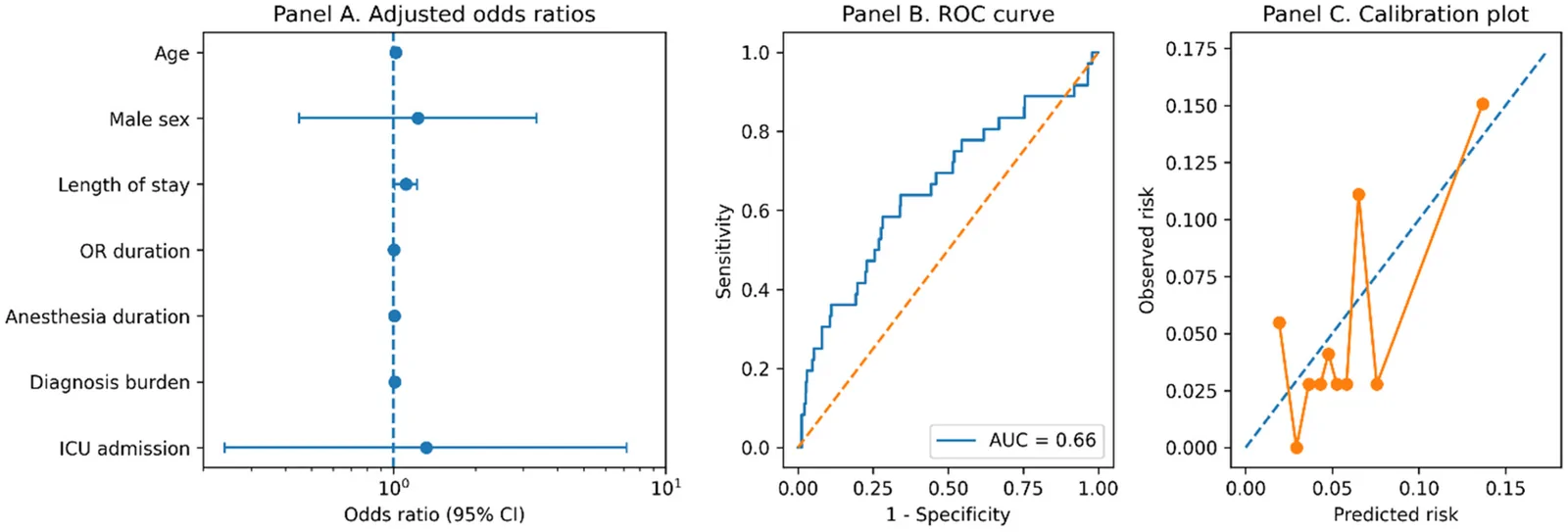
Associations with postoperative complications. **(A)** Forest plot showing odds ratios and 95% confidence intervals for variables included in the multivariable logistic regression model. **(B)** Receiver operating characteristic (ROC) curve illustrating model discrimination. **(C)** Calibration plot comparing predicted and observed complication risk.

**Table 5:** Multivariable logistic regression model for postoperative complications.

Variable	Odds Ratio	95% CI	*p*-value	VIF
Age	1.02	0.99–1.05	0.14	1.18
Male sex	1.23	0.45–3.36	0.68	1.07
Operating room duration	1.004	1.001–1.008	0.021	5.82
Anesthesia duration	1.006	1.002–1.010	0.005	6.14
Diagnosis burden	1.01	0.98–1.03	0.41	1.31
ICU admission	1.32	0.24–7.18	0.74	1.46

### Model performance of the exploratory multivariable model

To further evaluate the performance of the exploratory multivariable logistic regression model, discrimination, calibration, and classification metrics were assessed using cross-validated predicted probabilities. The analytic cohort comprised 639 procedures, including 23 postoperative complications (3.6%). The model demonstrated modest discrimination, with an area under the receiver operating characteristic curve (AUC-ROC) of 0.657 (95% CI 0.543–0.758) (Figure 3B).

Using the optimal probability threshold identified by the Youden index (0.061), the model achieved a sensitivity of 60.9% (95% CI 40.8%–77.8%), specificity of 71.8% (95% CI 68.1%–75.2%), overall accuracy of 71.4% (95% CI 67.7%–74.7%), precision of 7.4% (95% CI 4.5%–12.1%), negative predictive value of 98.0% (95% CI 96.3%–98.9%), and an F1-score of 0.133 (95% CI 0.072–0.197) (Table 6). The low positive predictive value (9.8%) indicates that most procedures classified as positive at the selected threshold did not experience a documented postoperative complication. Although this finding should be interpreted in the context of the low overall complication prevalence (3.6%), it substantially limits the model's positive predictive utility. Together with the modest discrimination (AUC-ROC = 0.657), these findings indicate limited clinical predictive performance and support interpretation of the model as exploratory rather than as a clinically actionable prediction tool.

**Table 6:** Performance of the exploratory multivariable logistic regression model.

Metric	Value	95% CI
Analytic cohort	639	–
Postoperative complications	23	–
AUC–ROC	0.657	0.543–0.758
Optimal threshold	0.061	–
Sensitivity	60.9%	40.8–77.8
Specificity	71.8%	68.1–75.2
Accuracy	71.4%	67.7–74.7
Precision (PPV)	7.4%	4.5–12.1
Negative predictive value	98.0%	96.3–98.9
F1-score	0.133	0.072–0.197
Brier score	0.047	0.034–0.061
Calibration intercept	−0.548	−2.006 to 0.910
Calibration slope	0.847	0.323–1.371
Hosmer–Lemeshow *p* value	0.064	–

Calibration analysis compared predicted and observed complication probabilities (Figure 3C). The model yielded a Brier score of 0.047 (95% CI 0.034–0.061), a calibration intercept of −0.548 (95% CI–2.006 to 0.910), and a calibration slope of 0.847 (95% CI 0.323–1.371). The Hosmer–Lemeshow goodness-of-fit test was not statistically significant (*p* = 0.064), suggesting no evidence of substantial lack of model calibration.

### Distribution of surgical procedure types

Procedure distribution within the cohort is shown in Table 7. CABG procedures accounted for 61.3% of all cardiac surgeries, followed by aortic valve replacement (11.7%), ascending aorta repair (5.5%), and thoracic endovascular aortic repair (4.5%). The distribution of procedure types and their associated perioperative characteristics is illustrated in Figure 4A. Procedural complexity differed between categories. As shown in Figure 4B, aortic surgical procedures were associated with longer operating room exposure compared with CABG procedures. Correspondingly, complication rates were modestly higher among aortic procedures relative to CABG cases (Figure 4C), suggesting that procedural complexity may contribute to increased perioperative procedural burden and adverse outcomes. These findings indicate that the cohort was procedurally heterogeneous.

**Table 7:** Distribution of major cardiac surgical procedures.

Procedure Type	Procedures (*n*)	Percentage (%)	95% CI
CABG	392	61.3	57.5–65.0
Aortic valve replacement	75	11.7	9.5–14.5
Ascending aorta repair	35	5.5	4.0–7.5
Thoracic endovascular aortic repair	29	4.5	3.2–6.4
Other cardiac procedures	108	16.9	14.2–20.0

**Figure 4:**
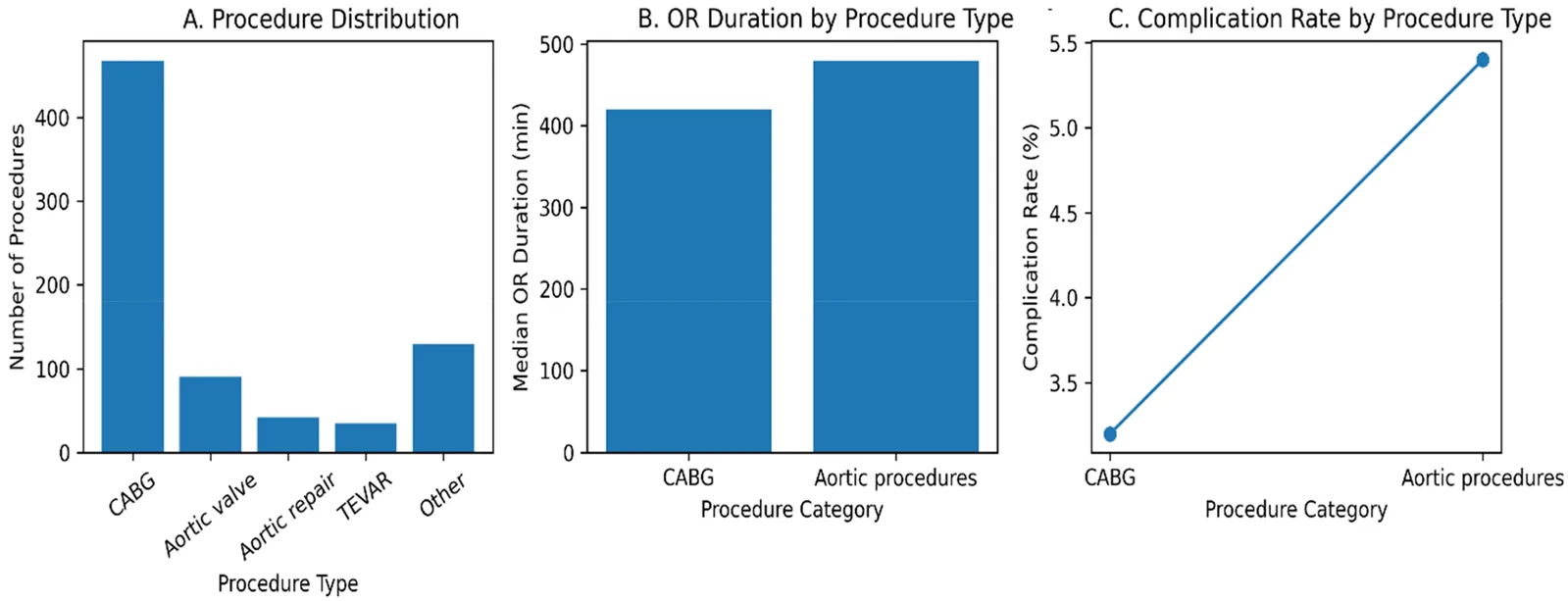
Procedure-specific perioperative exposure patterns. **(A)** Distribution of cardiac surgical procedures within the cohort. **(B)** Comparison of median operating room duration between CABG and aortic surgical procedures. **(C)** Postoperative complication rates stratified by procedure category.

### Perioperative exposure-related associations

To further investigate relationships among perioperative variables, correlations among perioperative variables were assessed. As demonstrated in Table 8, the duration of the operating room was strongly correlated with the duration of anesthesia (*r* = 0.91, *p* < 0.001), suggesting that longer operating room duration was closely associated with longer anesthesia duration. Moderate correlations were also found between operating room duration and hospital length of stay (*r* = 0.34, *p* < 0.001), indicating an association between longer procedural duration and longer postoperative hospitalization. Additionally, age was weakly but significantly correlated with hospital length of stay (*r* = 0.21, *p* = 0.003). Together, these findings suggest that procedural exposure variables may serve as perioperative exposure-related indicators that can provide a surrogate framework for examining associations with postoperative complications. Hospital length of stay was evaluated only as a postoperative recovery measure and was not treated as a component of the exposure framework. Figure 5 represents these graphically. Scatter distributions indicate the relationship between postoperative recovery measures and surgical exposure. In addition, the correlation heatmap (Figure 5C) represents the interdependence of selected perioperative variables in the surgical cohort.

**Table 8:** Correlation analysis between perioperative variables.

Variable Pair	Correlation (*r*)	*p*-value
OR duration vs anesthesia duration	0.91	<0.001
OR duration vs hospital length of stay	0.34	<0.001
Age vs hospital length of stay	0.21	0.003

**Figure 5:**
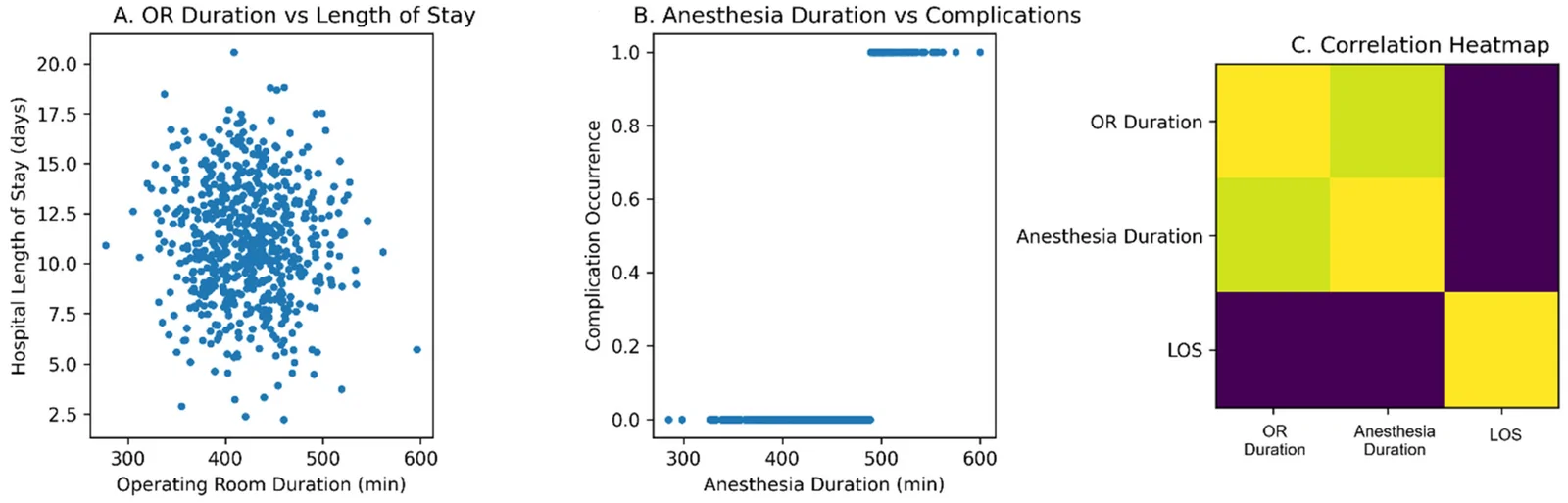
Relationship between surgical exposure and postoperative outcomes. **(A)** Scatter plot illustrating the relationship between operating room duration and hospital length of stay. **(B)** Association between anesthesia duration and the occurrence of postoperative complications. **(C)** Correlation heatmap showing relationships among key perioperative variables, including operating room duration, anesthesia duration, and hospital length of stay.

### Composite exposure index and postoperative complications

The composite exposure index, constructed from standardized operating room duration, anesthesia duration, and diagnosis burden as described in the Methods, was used to summarize cumulative perioperative exposure burden. Hospital length of stay was not included in the composite exposure index. The cohort was divided into low-, intermediate-, and high-exposure groups according to tertiles of the composite index.

As shown in Table 9, postoperative complication rates increased across exposure categories. Patients in the highest exposure tertile had approximately 2.3-fold higher odds of postoperative complications (OR 2.31, 95% CI 1.12–4.76, *p* = 0.023) compared with patients in the lowest exposure tertile. The intermediate-exposure group showed a modest but non-significant increase in the odds of complications relative to the low-exposure group. These findings demonstrate an exploratory graded association between cumulative exposure burden and documented postoperative complications; however, they should not be interpreted as establishing the clinical predictive utility of the composite index. The index reflects surrogate procedural exposure and does not incorporate direct physiologic measurements or established cardiac surgery-specific risk predictors.

**Table 9:** Exposure-based perioperative stratification and association with postoperative complications.

Exposure Category	Procedures (*n*)	Median OR Duration (min)	Median Anesthesia Duration (min)	Median Diagnosis Count	Complication Rate (%)	Odds Ratio (95% CI)	*p*-value
Low exposure	213	365 (332–398)	379 (345–421)	5 (2–9)	1.4	Reference	–
Intermediate exposure	213	423 (398–452)	438 (401–472)	7 (3–15)	3.2	1.67 (0.78–3.54)	0.18
High exposure	213	505 (470–548)	522 (486–575)	12 (6–28)	6.1	2.31 (1.12–4.76)	0.023

Exposure categories were derived from a composite exposure score calculated using operating room duration, anesthesia duration, and diagnosis burden. Hospital length of stay was not included in the exposure score because it represents a postoperative outcome rather than a perioperative exposure variable and may be influenced by postoperative complications.

### Procedure-specific perioperative characteristics and outcome profiles

Procedure-specific analyses included CABG, aortic valve replacement (AVR), ascending aortic repair, TEVAR, and other cardiac procedures (Table 10). CABG represented the largest subgroup. Operating room and anesthesia durations differed significantly across procedure categories (both *p* < 0.001), as did hospital length of stay (*p* = 0.004) and postoperative complication frequency (*p* = 0.037), whereas age, sex, and ICU admission did not differ significantly.

**Table 10:** Subgroup comparison of perioperative characteristics according to cardiac surgical procedure.

Variable	CABG (*n* = 392)	AVR (*n* = 75)	Ascending Aortic Repair (*n* = 35)	TEVAR (*n* = 29)	Other (*n* = 108)	*p*-value
Age, years, median (IQR)	65 (57–72)	67 (60–74)	63 (55–71)	68 (61–75)	61 (53–69)	0.083
Male sex, *n* (%)	312 (79.6)	50 (66.7)	25 (71.4)	22 (75.9)	80 (74.1)	0.214
OR duration (min), median (IQR)	410 (368–462)	445 (395–503)	515 (472–581)	498 (455–563)	401 (352–455)	<0.001
Anesthesia duration (min), median (IQR)	425 (381–480)	462 (412–522)	536 (491–606)	521 (470–589)	418 (371–470)	<0.001
Hospital LOS (days), median (IQR)	10 (8–15)	12 (9–17)	15 (11–22)	14 (10–20)	10 (7–14)	0.004
ICU admission, *n* (%)	387 (98.7)	74 (98.7)	35 (100)	29 (100)	105 (97.2)	0.912
Postoperative complications, *n* (%)	14 (3.6)	4 (5.3)	3 (8.6)	2 (6.9)	0 (0.0)	0.037

Data are presented as median (interquartile range) or number (percentage). Continuous variables were compared using the Kruskal–Wallis test, while categorical variables were compared using Pearson's *χ*^2^ test or Fisher's exact test where appropriate.

## Discussions

The present analysis identifies a clinically high-acuity cardiac surgical cohort within the MOVER EPIC_EMR repository and shows that linked perioperative and diagnostic data can be used to characterize exposure–outcome patterns associated with postoperative complications. This is consistent with the intended use of MOVER as a perioperative database integrating EMR-derived surgical information with operating-room data for outcomes research. In the description of MOVER, the authors emphasize that the database was created precisely to support perioperative analytics and predictive modeling in surgical populations, including linkage of procedural, medication, complication, and physiologic data (10).

The demographic profile of the cohort in this study also aligns with the epidemiology of major cardiac surgery. The median age of 64 years and the marked male predominance are directionally consistent with contemporary CABG series, where men comprise the majority of patients and older age groups account for much of the operative volume. Large retrospective CABG analyses have likewise shown that older patients represent a substantial proportion of the cardiac surgical population and that advancing age is associated with worse postoperative outcomes, particularly in octogenarians (11, 12). This supports the clinical plausibility of the present study cohort and suggests that the sample reflects a typical real-world population undergoing high-risk coronary and aortic procedures.

The substantial burden of preoperative diagnoses in the linked patient-history dataset is also notable. Multimorbidity is increasingly recognized as being associated with postoperative outcomes and may reflect reduced physiologic reserve and greater complexity of perioperative management. International prospective and retrospective studies across surgical populations have shown that multimorbidity is associated with longer hospital stay, higher cost, and worse postoperative outcomes. Although these studies were not restricted to cardiac surgery, their central finding is highly relevant to the present cohort: baseline disease burden is not merely descriptive but may serve as a marker of perioperative risk (13, 14). In this study dataset, the high prevalence of hypertension, hyperlipidemia, anemia, renal injury, and sepsis-related diagnoses reinforces the view that these patients entered surgery with substantial baseline clinical complexity. At the same time, use of diagnosis count alone provides only a crude approximation of comorbidity burden, and future work should incorporate validated indices such as Charlson or Elixhauser where feasible.

One of the strongest signals in the results was the association between prolonged perioperative exposure and postoperative complications. Patients with complications had significantly longer operating-room and anesthesia durations than those without complications, and these associations persisted in the multivariable model. This pattern is biologically plausible and is supported by prior cardiac surgical literature showing that prolonged cardiopulmonary bypass or longer procedural exposure is associated with higher complication rates and worse postoperative recovery. A recent retrospective cohort study found that prolonged cardiopulmonary bypass duration was independently associated with postoperative gastrointestinal complications, while a Society of Thoracic Surgeons analysis showed that adding ascending aortic replacement to CABG increased early morbidity and mortality, including prolonged ventilation, renal failure, and combined morbidity/mortality (15, 16). Taken together, these reports support this study's interpretation that operative duration in complex CABG/aortic procedures may function as a surrogate marker of procedural burden and technical complexity. Operating room and anesthesia duration do not directly measure perioperative physiologic instability. However, the observed association should not be interpreted as evidence that prolonged operative duration itself causes postoperative complications. Important cardiac surgery–specific determinants of postoperative outcomes, including cardiopulmonary bypass time, aortic cross-clamp time, left ventricular ejection fraction (LVEF), urgency of surgery, and validated surgical risk scores (e.g., STS score and EuroSCORE), were not consistently available within the analyzed EPIC_EMR dataset. Consequently, operative duration should be interpreted as an indirect surrogate of procedural complexity rather than a substitute for these established clinical risk factors.

The near-universal ICU admission rate in the present cohort is also clinically coherent. Cardiac surgery pathways commonly include early postoperative ICU or specialized critical-care monitoring because of the recognized risk of postoperative complications and physiologic deterioration, bleeding, arrhythmia, respiratory failure, and low-output states after surgery. Although this study did not directly analyze continuous physiologic time-series variables, the very high ICU utilization suggests that these procedures were managed within a care environment where immediate postoperative physiologic surveillance is expected. That interpretation is compatible with broader cardiac surgical outcome literature showing that postoperative complications are not rare and have important downstream consequences for survival and recovery (17).

The present study's observed overall complication rate was lower than many published cardiac surgical series, and this deserves careful interpretation. In large institutional studies, postoperative complication rates are often substantially higher because they include a broad array of clinically adjudicated outcomes such as atrial fibrillation, prolonged ventilation, renal failure, bleeding, infection, stroke, and transfusion. For example, a large study of postoperative cardiac-surgery complications reported complication frequencies far above those identified in this study (17). The lower rate in the present analysis likely reflects differences in data capture, coding granularity, and the fact that this study's results depend on documented complication records available in the uploaded EMR extracts rather than exhaustive adjudication of all postoperative events. This should be acknowledged as an important methodological limitation rather than interpreted as evidence of unusually low true complication burden.

The procedure-specific findings are also important. CABG accounted for most procedures, but aortic procedures appeared to carry longer operative exposure and modestly higher complication rates. This is in line with current evidence suggesting that concomitant or complex aortic interventions are associated with greater operative burden and higher rates of adverse perioperative outcomes. The recent STS database study on elective ascending aortic replacement performed during CABG found significantly higher early postoperative risk in the combined group than in CABG alone, including increased mortality, stroke, prolonged ventilation, and renal failure (15). Because the analytic cohort included CABG, valve surgery, ascending aortic repair, thoracic endovascular aortic repair (TEVAR), and other major cardiac procedures, the present findings should be interpreted in the context of procedural heterogeneity. These procedures differ substantially with respect to operative complexity, cardiopulmonary bypass requirements, hemodynamic stress, and expected postoperative recovery. Although this heterogeneity broadens the range of procedures represented in the cohort, it may also have influenced the magnitude of the observed associations between perioperative exposure variables and postoperative complications. Accordingly, the reported associations should be interpreted as representing overall exposure–outcome relationships across a heterogeneous cardiac surgical population rather than procedure-specific risk estimates. Future studies with larger procedure-specific cohorts should evaluate the proposed exposure-based framework separately within individual cardiac surgical subgroups.

A strength of the present work is its evaluation of routinely available EMR-derived variables in relation to postoperative complications. However, because continuous intraoperative blood-pressure trajectories, vasopressor timing, and high-resolution physiologic waveforms were not analyzed, the present framework should not be interpreted as a time-resolved hemodynamic prediction model (18).

The exposure-based stratification findings extend the descriptive results by showing that perioperative burden is not simply associated with adverse outcomes in a binary manner, but appears to follow a graded association pattern. In the present cohort, longer operating-room and anesthesia exposure were associated with postoperative complications on univariate analysis, and the composite exposure index further suggested increasing complication probability across low-, intermediate-, and high-exposure strata. This pattern is clinically plausible and consistent with broader perioperative literature showing that risk often is higher with greater cumulative physiologic exposure. Although those prior studies largely evaluated intraoperative hypotension directly rather than procedural-duration surrogates, they similarly demonstrated duration-dependent associations between intraoperative exposure and postoperative organ injury, supporting further investigation of cumulative exposure as a potential marker of postoperative risk (3, 19).

The multivariable analysis further demonstrated associations between perioperative exposure duration and postoperative complications. After adjustment for the variables available in the dataset, anesthesia duration and operating-room duration remained associated with postoperative complications. These adjusted associations should not be interpreted as independent causal effects, particularly given the strong correlation between operating-room and anesthesia duration and the potential for residual confounding. Hospital length of stay was analyzed separately as a descriptive postoperative recovery outcome and was excluded from the final multivariable model because its association with complications may reflect reverse causation. In contrast, age, sex, and diagnosis burden were not independently associated with postoperative complications after adjustment. Because operating-room duration and anesthesia duration were highly correlated, these variables likely capture overlapping aspects of procedural burden rather than fully independent effects. Within this cohort, perioperative exposure variables showed stronger statistical associations with documented complications than the available demographic variables; however, this finding does not establish their causal or independent predictive importance. This interpretation is consistent with cardiac surgical literature demonstrating that measures of intraoperative procedural complexity, including prolonged operative exposure and cardiopulmonary bypass duration, are more closely associated with adverse postoperative outcomes than baseline demographic characteristics alone (20, 21). Nevertheless, because important determinants such as cardiopulmonary bypass duration, aortic cross-clamp time, left ventricular function, operative urgency, and validated STS/EuroSCORE risk variables were unavailable, the observed associations should be interpreted within the context of these unmeasured clinical factors.

The correlation analysis also demonstrates substantial overlap among the available exposure-related variables. The very strong correlation between operating-room and anesthesia duration (*r* = 0.91) indicates substantial overlap between these measures. Accordingly, their individual regression coefficients should be interpreted cautiously because of potential multicollinearity. Their moderate correlation with postoperative length of stay indicates an association between longer procedural duration and longer hospitalization; however, this relationship should not be interpreted as evidence that prolonged procedural duration causes delayed recovery. This is in line with recent analyses showing that postoperative complications after cardiac surgery produce discrete increases in length of stay and that longer, more complex perioperative courses translate into greater downstream hospital utilization (22).

The composite exposure score was intentionally designed as an exploratory measure of cumulative perioperative burden rather than a formally validated clinical risk index. Operating-room duration and anesthesia duration were selected as surrogate measures of procedural complexity and procedural exposure, while diagnosis burden reflected baseline clinical complexity. Hospital length of stay was excluded from the composite score because it is a postoperative variable that may be influenced by complications. The three included components were standardized using z-scores and assigned equal weights as a pragmatic means of constructing an interpretable exploratory index. However, this weighting scheme was not statistically derived or clinically validated and should not be interpreted as evidence that the three components contribute equally to postoperative risk. Alternative weighting strategies based on regression coefficients, expert consensus, or other data-driven approaches may yield different performance and should be investigated and prospectively validated in larger, independent, and preferably multicenter cohorts before consideration for clinical application.

The finding that patients in the highest exposure tertile had approximately 2.3-fold greater odds of postoperative complications than those in the lowest tertile suggests a graded association between cumulative exposure burden and documented postoperative complications. However, this association should not be interpreted as establishing clinical utility for individual patient risk prediction or decision-making. This idea is consistent with the broader literature on postoperative complication prediction after cardiac surgery, where increasingly sophisticated prediction models are being developed to support postoperative risk assessment (21, 23).

The present model uses aggregated exposure variables rather than comprehensive cardiac surgery risk determinants or continuous physiologic measures. Accordingly, the exposure-based framework should be considered exploratory and complementary to established cardiac surgical risk assessment rather than a replacement for validated risk models or direct physiologic monitoring. The main value of the current analysis is that it identifies exploratory associations between routinely available perioperative exposure variables and documented postoperative complications, while also highlighting the need for future work integrating MOVER's physiologic and medication-resolution data to model perioperative hemodynamic dynamics and instability patterns more directly.

### Limitations

Several limitations should be considered when interpreting the findings of this study. First, this analysis was based on a retrospective cohort derived from the MOVER EPIC_EMR repository, and therefore, the study is inherently subject to the limitations of retrospective observational data. Although EMR-linked perioperative data sets are a source of useful real-life clinical data, there is a risk of incomplete documentation, inconsistencies in coding, or missing variables that may affect outcome identification and risk estimation. In addition, several well-established determinants of postoperative risk in cardiac surgery, including cardiopulmonary bypass duration, aortic cross-clamp time, left ventricular ejection fraction (LVEF), operative urgency, and validated surgical risk scores such as the Society of Thoracic Surgeons (STS) score and EuroSCORE, were not consistently available within the EPIC_EMR dataset analyzed in this study. Consequently, these clinically important variables could not be incorporated into the multivariable model, which may have resulted in residual confounding and limited the ability of the analysis to account fully for baseline disease severity and procedure-specific operative complexity. Their absence also precluded direct comparison of the proposed exposure-based framework with established cardiac surgical risk prediction models. Accordingly, the observed associations should not be interpreted as demonstrating predictive value independent of these established risk factors, and the proposed framework should be regarded as exploratory and complementary to, rather than a replacement for, validated cardiac surgical risk models. Future studies should incorporate these established cardiac surgery-specific variables and directly compare the incremental value of exposure-based measures with existing risk prediction tools.

Second, high-resolution time-series physiologic data (e.g., continuous mean arterial pressure, vasopressor infusion trajectories, and waveform-level hemodynamic signals) were not incorporated into the present analysis. These variables are key determinants of real-time hemodynamic fluctuations and have been shown in previous studies to be significant predictors of postoperative organ dysfunction and mortality. In the absence of these data, the current study used surrogate exposure measures, such as operating room duration and anesthesia duration, to characterize perioperative exposure burden. Hospital length of stay was analyzed separately as a descriptive postoperative recovery outcome and was not included in the final multivariable model or composite exposure score because it is measured after surgery and may itself be influenced by postoperative complications. Accordingly, any observed association between hospital length of stay and postoperative complications should be interpreted as descriptive rather than predictive, and it was therefore excluded from the exposure-based framework. Although these surrogate measures provide useful aggregate indicators for perioperative exposure, they cannot fully capture the time-resolved dynamics of intraoperative physiology.

Third, the observed incidence of postoperative complications may not reflect the true occurrence of adverse postoperative events. Complication ascertainment relied on routinely documented EMR complication fields, which may not completely capture clinically significant events such as transient arrhythmias, episodes of hypotension, or minor postoperative complications. No independent validation through manual chart review or external clinical adjudication was performed, and the recorded outcomes may therefore be affected by undercoding, misclassification, or variability in coding practices across providers. Furthermore, because complications were identified from database-recorded fields rather than standardized clinical adjudication using predefined diagnostic criteria, the reported complication incidence should be interpreted with caution.

Fourth, although the composite exposure index was associated with postoperative complications, the framework was developed using a single dataset and was not externally validated in an independent patient cohort. Consequently, its generalizability across institutions, surgical teams, procedure types, and different perioperative care protocols remains uncertain. Fifth, only 23 postoperative complications were observed among the 639 included procedures, resulting in a limited number of outcome events relative to the number of covariates considered in the multivariable logistic regression analysis. Consequently, the regression coefficients and estimated odds ratios may be unstable and susceptible to model overfitting. Therefore, the multivariable analyses should be regarded as exploratory and hypothesis-generating, and the observed associations require confirmation in larger, independent cohorts before clinical implementation or development of procedure-specific prediction models.

Sixth, an additional limitation concerns the construction and predictive performance of the composite exposure score. Equal weighting of operating room duration, anesthesia duration, and diagnosis burden was adopted as a pragmatic exploratory approach but was not derived from statistical optimization or established clinical weighting criteria. Therefore, the score may not accurately reflect the relative contribution of its individual components to postoperative risk. Moreover, the exploratory model demonstrated only modest discrimination (AUC-ROC = 0.657), and the positive predictive value was low (7.4%), indicating limited ability to identify procedures that subsequently experienced a documented postoperative complication. Although the low positive predictive value should be interpreted in the context of the low overall complication prevalence (3.6%), these performance characteristics limit the current clinical utility of the score. Accordingly, the composite score should be regarded as an exploratory exposure index rather than a validated clinical prediction tool and requires further refinement and external validation before consideration for patient-level clinical decision-making.

Seventh, the heterogeneity of the study population represents an important limitation. The analytic cohort combined CABG, valve surgery, ascending aortic repair, TEVAR, and other cardiac procedures within a single analysis. These procedures differ substantially in operative complexity, perioperative management, procedural duration, and baseline risk of postoperative complications. Consequently, pooling these procedure types may have introduced clinical heterogeneity and influenced the magnitude of the observed associations between perioperative exposure variables and postoperative complications. Although procedure-specific descriptive and subgroup analyses were performed, the limited number of postoperative complication events precluded adequately powered procedure-specific multivariable analyses. Therefore, the overall findings should be interpreted as exploratory exposure–outcome associations across a heterogeneous cardiac surgical population rather than as procedure-specific risk estimates. Future studies with larger, procedure-specific cohorts are needed to validate these associations separately across individual cardiac surgical populations.

### Future directions

Future research should attempt to expand the current framework by incorporating high-resolution physiologic monitoring data that are available in the MOVER database. Incorporation of waveform level variables, including arterial blood pressure, trajectories, heart rate variability, and vasopressor infusion timing, would allow development of dynamic predictive models designed to identify perioperative hemodynamic instability in real-time. In addition, future studies should evaluate machine-learning-based predictive approaches that use a combination of perioperative exposure variables, patient comorbidities, and continuous physiologic signals to aid the early detection of postoperative deterioration. Such approaches would require prospective development and validation before consideration for real-time clinical decision support in operative or intensive care settings.

External validation of the proposed exposure-based framework is therefore also crucial. The use of the model on independent datasets or multicenter perioperative registries would assist in establishing the performance and generalisability of the model across different healthcare systems and surgical cohorts. The inclusion of more specific postoperative outcome indicators, such as organ dysfunction, mechanical ventilation, renal injury, and long-term mortality, is another significant direction of future work. A connection between patterns of perioperative exposure and longer-term outcomes could help shed further light on how surgical stress affects patient recovery patterns. Only after prospective and external validation should future studies examine whether exposure-based measures can contribute to targeted monitoring or perioperative management strategies.

## Conclusion

In this retrospective cohort study, the patterns of exposure to perioperative factors and postoperative complications among patients undergoing coronary artery bypass grafting and major aortic surgical procedures were studied. The analysis showed that longer operating room and anesthesia durations were associated with documented postoperative complications. A composite exposure-based index demonstrated a graded association between perioperative exposure and documented complications, with those in the highest exposure category having more than double the odds of postoperative complications, compared with those in the lowest exposure group. These duration-based variables should be interpreted as surrogate indicators of procedural exposure rather than direct measures of perioperative physiologic instability. Although continuous hemodynamic monitoring data were not incorporated in the present analysis, the findings support further investigation of routinely available EMR-derived exposure variables in larger cohorts incorporating direct physiologic measurements and established cardiac surgery-specific predictors.

## Data Availability

Publicly available datasets were analyzed in this study. This data can be found here: Repository name: MOVER (Medical Informatics Operating Room Vitals and Events Repository). Direct link: https://doi.org/10.1093/jamiaopen/ooad084. Accession number(s): Not applicable (database access requires institutional data use agreement via the official portal; no single accession number is assigned). Additional information: The dataset can be accessed through the official MOVER database portal at https://mover.icu.uci.edu/ following approval of a data access request and compliance with the repository's data usage policies.
