# Supplementary material for: Exposure-based stratification of postoperative complications in major cardiac surgical procedures: a retrospective cohort study using the MOVER database
Source: Front Cardiovasc Med. 2026 Sep 21;13:1887472. doi: 10.3389/fcvm.2026.1887472 (PMC13634841; doi:10.3389/fcvm.2026.1887472)
Supplement: Supplementary file 1 [file Table1.docx]

**Table S1. Types and frequencies of documented postoperative complications**

| **Complication category** | | | **No. of procedures, n** | **% of total cohort (N=639)** | **% of complicated procedures (n=23)** |
| --- | --- | --- | --- | --- | --- |
| Cardiovascular complications | Circulatory/hemodynamic-related events | | 4 | 0.63 | 17.39 |
|  | Other cardiovascular events  Other cardiovascular events | Arrhythmia-related event | 1 | 0.16 | 4.35 |
|  |  | Myocardial ischemic/cardiac event | 1 | 0.16 | 4.35 |
|  |  | Other cardiac event | 1 | 0.16 | 4.35 |
| Non-cardiovascular complications | Respiratory/pulmonary | | 5 | 0.78 | 21.74 |
|  | Renal | | 3 | 0.47 | 13.04 |
|  | Bleeding/hematologic | | 3 | 0.47 | 13.04 |
|  | Neurologic | | 2 | 0.31 | 8.70 |
|  | Infectious | | 2 | 0.31 | 8.70 |
|  | Metabolic/other systemic | | 1 | 0.16 | 4.35 |
| Total procedures with ≥1 postoperative complication | | | 23 | 3.60 | 100.00 |
